# Differentiated Thyroid Cancer in a Pediatric Population: Estimating the Risk of Recurrence and Evolution Over Time

**DOI:** 10.7759/cureus.34313

**Published:** 2023-01-28

**Authors:** Inês Damásio, Daniela Dias, Sara Pinheiro, Susana Esteves, Valeriano Leite, Rita Santos

**Affiliations:** 1 Endocrinology, Instituto Português de Oncologia de Lisboa Francisco Gentil, Lisboa, PRT; 2 Endocrinology, Hospital CUF Descobertas, Lisboa, PRT; 3 Endocrinology, Diabetes and Metabolism, Instituto Português de Oncologia de Lisboa Francisco Gentil, Lisboa, PRT; 4 Clinical Research, Instituto Português de Oncologia de Lisboa Francisco Gentil, Lisboa, PRT; 5 Endocrinology, Hospital CUF Cascais, Lisboa, PRT

**Keywords:** follow-up, guidelines, dynamic risk stratification, pediatric age, differentiated thryroid carcinoma

## Abstract

Background

Differentiated thyroid cancer (DTC) is the most common endocrine cancer during childhood, and the prognosis is usually good. The 2015 American Thyroid Association (ATA) pediatric guidelines for DTC classify patients into three categories (low, intermediate, and high) that represent the risk for persistent/recurrent disease. The "Dynamic Risk Stratification" (DRS) System showed that, in adults, reassessment of disease status during follow-up was a better predictor of disease status at the end of follow-up when compared to ATA risk stratification. This system is still not validated for the pediatric population with DTC. Our aim was to evaluate the usefulness of the DRS system in predicting DTC disease behaviour in this specific population. We also aimed to evaluate potential clinical-pathological factors associated with persistent disease at the end of follow-up.

Methods

A retrospective analysis of 39 pediatric patients (≤18 years) with DTC was conducted in our institution between 2007 and 2018, including 33 patients who had follow-up ≥ 12 months; these were classified into ATA risk groups and re-stratified according to their response to treatment at 12-24 months of follow-up. The associations between the ordinal variables of the baseline ATA risk group and the disease status re-evaluated 12-24 months after diagnosis (as per the DRS system) and at the end of follow-up were evaluated using a linear-by-linear association test. Gender, age at diagnosis, tumor size, multicentricity, extrathyroid extension, vascular invasion, lymph node metastasis, distant metastasis, and stimulated thyroglobulin (sTg) during the first RAI administration were evaluated as potential factors associated with persistent disease at 27 months after diagnosis using Firth's bias-reduced penalized-likelihood logistic regression.

Results

In this study, 39 patients were retrospectively analyzed, including 33 patients who had follow-ups ≥ 12 months with a median time of 56 (27-139) months who were classified in ATA risk groups and then re-stratified depending on their response to treatment between 12 and 24 months of follow-up. There was a statistically significant association between ATA risk groups and re-evaluation at 12 and 24 months (p=0.001) and between these two stratifications and the state of disease at final follow-up (p<0.001 for both). Factors with a statistically significant association with persistent disease at 27 months of follow-up were male sex, lymph node metastases at diagnosis, distant metastasis, extrathyroidal extension, and stimulated Tg values.

Conclusions

The assessment of the response to treatment between 12 and 24 months and at the end of follow-up refines the initial ATA risk stratification, confirming that dynamic risk evaluation is also helpful in the pediatric population.

## Introduction

Differentiated thyroid carcinoma (DTC) is an increasingly prevalent entity among children and adolescents. Papillary thyroid carcinoma (PTC) is the most frequent histological type [1]. Compared to adults, children with PTC are more likely to have regional lymph node involvement, extrathyroidal extension, and pulmonary metastases. However, despite more extensive disease at presentation, children have lower disease-related mortality (≤2%) and a more favorable progression-free survival. Even 30-45% of children with metastatic pulmonary disease tend to have persistent but stable disease after radioiodine (RAI) therapy [2]. The more recent ATA Guidelines [2] concerning DTC in children recommend an initial stratification into three groups (low, intermediate, and high-risk), according to the risk of recurrent/persistent disease based on histopathological data. However, this is a static representation that does not consider the response to treatment which may greatly impact the disease's clinical course. Tuttle et al. [3] designed the Dynamic Risk Stratification (DRS) system, where the risk of recurrent/persistent disease in patients with DTC was reassessed during follow-up according to the response to initial therapy, determined by clinical, biochemical, and imaging data. This system was validated only for the adult population with DTC, although some studies investigate its use in children [4-7]. Our aims were to access the performance of the DRS system in the pediatric population followed in our centre and to evaluate potential clinical-pathological factors associated with persistent disease.

## Materials and methods

Patients

A total of 53 pediatric patients (age≤18 years) with histological diagnoses of DTC were followed in our institution between 2007 and 2018 and were retrospectively analyzed; 10 patients were excluded because they were lost to follow-up. Four patients with noninvasive follicular thyroid neoplasm with papillary-like nuclear features were also excluded. The remaining 39 patients were included in the descriptive analysis as well as the 33 patients who had follow-up ≥ 12 months and were classified in ATA risk groups and re-stratified according to their response to treatment at 12-24 months of follow-up. The median time of follow-up in this group was 56 months (range: 27-139 months) and the median re-evaluation time was 21 months.

Treatment protocol and follow-up

The treatment protocol included surgery (lobectomy, total thyroidectomy with or without lymphadenectomy, and removal of a thyroglossal cyst), with or without administration of one ablative or therapeutic RAI, as indicated, followed by levothyroxine therapy. The dosage of levothyroxine therapy was decided to target specific thyroid-stimulating hormone (TSH) values according to the initial risk stratification. The length of follow-up was defined as the time between the first surgery and the last medical visit. The management protocol included clinical assessment three months after surgery with physical examination and measurement of serum thyroglobulin (Tg) levels under thyrotropin (TSH) suppression and antithyroglobulin antibodies (TgAB). A neck ultrasound was performed in the first year of follow-up. Tg and TgAB were measured periodically with a mean interval of six months. Patients classified as disease-free had annual evaluations, while those with persistent disease were evaluated more often with additional diagnostic imaging as indicated.

ATA risk stratification

The 33 patients with follow-up periods longer than 12 months were initially stratified in accordance with the ATA Guidelines [2] in three categories (low, intermediate, and high-risk), shown in Table 1.

**Table 1:** Definition of ATA risk groups AJCC: American Joint Committee on Cancer; ATA: American Thyroid Association; DTC: differentiated thyroid carcinoma Definitions of minimal N1a and N1b disease were adopted from the ATA Surgical Affairs Committee’s taskforce on thyroid cancer nodal surgery [8]

ATA Risk groups	Definition
ATA low-risk group	DTC confined to the thyroid (eight AJCC/UICC TNM staging system T1-T3) with clinical N0/Nx or with incidental pathological micrometastases pN1a* (≤ 5 lymph nodes with the largest dimension of metastatic focus <0,2 cm) and with no distant metastasis (M0).
ATA intermediate-risk group	Extensive pN1a (> 5 lymph nodes or largest dimension of metastatic focus ≥ 0,2 cm) or minimal N1b* disease (defined as <10 lymph nodes or largest dimension of metastatic focus <3 cm) and with no distant metastasis (M0).
ATA high-risk group	Regionally extensive disease (pN1b with >10 lymph nodes or largest dimension of metastatic focus ≥ 3 cm), or with locally invasive disease (T4) or distant metastasis.

DRS and status at the end of follow-up

The revaluation of clinical status was done between 12-24 months after diagnosis. It relied on the measurement of Tg under suppression, neck ultrasound, and other imaging modalities performed in patients with persistent disease. Table 2 shows the definitions of DRS and status at the end of the follow-up.

**Table 2:** Definition of response at 12-24 months follow-up and at the end of follow-up BED: biochemical evidence of disease; NED: no evidence of disease; RAI: radioiodine, SED: structural evidence of disease; TgAb: antithyroglobulin antibodies; Tg: thyroglobulin; TSH: thyroid-stimulating hormone The criteria in the table has been adapted from Sung et al. [7]

Response at 12-24 months	Status at the end of follow-up	Total thyroidectomy with or without RAI treatment	Lobectomy alone*
Excellent response	NED	Negative imaging and suppressed Tg < 0,2 ng/mL or stimulated Tg < 1 ng/mL, undetectable TgAb	Negative imaging and stable nonstimulated Tg < 30 ng/mL
Indeterminate response	Indeterminate state	Nonspecific imaging findings and suppressed Tg 0.2-1 ng/mL or TgAb stable or declining	TgAb stable or declining and no evidence of structural disease
Biochemical incomplete response	BED	Negative imaging and suppressed Tg ≥ 1 ng/mL or stimulated Tg ≥ 10 ng/mL or TgAb rising	Negative imaging and nonstimulated Tg > 30 ng/mL or increasing Tg Levels with similar TSH levels or TgAb rising
Structural incomplete response	SED	Structural or functional evidence of disease with any Tg or TgAbs levels	Structural or functional evidence of disease with any Tg or TgAbs levels

Stimulated Tg

In patients that required RAI treatment (RAIT), we measured stimulated Tg (sTg) just before the administration of RAI. TSH at that time was considered appropriate if >30 mIU/L in patients with RAIT performed in hypothyroidism and >100 mIU/L when RAIT was performed under recombinant-human (rh) TSH. RAIT was administered with a median of two months (range 1-8 months) after surgery, and the median RAI activity used was 60 mCi (30-119 mCi). Patients with positive interfering Tg- antibodies (n=11) were excluded from the analysis regarding sTg.

Statistical analysis

Clinical and demographic data are reported as median, percentiles 25 and 75 (P25-75, interquartile range) and minimum and maximum for continuous variables, and relative and absolute frequencies for categorical variables. The associations between the ordinal variables baseline ATA risk group (ordered as low, intermediate, and high) and the disease status re-evaluated 12-24 months after diagnosis (as per DRS system) and at the end of follow-up (both ordered as no evidence of disease (NED), indeterminate, biochemical evidence of disease (BED), and structural evidence of disease (SED)) were evaluated using a linear-by-linear association test considering the approximation of the null distribution of the test statistic via Monte Carlo resampling. We also calculated Somers’ D, an asymmetric nonparametric measure of the strength and direction of association between ordinal variables. Gender, age at diagnosis, tumor size, multicentricity, extrathyroid extension, vascular invasion, lymph node metastasis, distant metastasis, and sTg during the first RAI administration were evaluated as potential factors associated with persistent disease at 27 months after diagnosis, which was the minimum follow-up for all patients, using Firth's bias-reduced penalized-likelihood logistic regression. The patients with indeterminate disease status at the end of follow-up were excluded from this analysis. The small sample size and the small number of cases with persistent disease (only nine patients) precluded a multivariable analysis. ROC analysis was used to determine optimal cut-offs of sTg that could better separate patients with persistent disease from patients in remission. All statistical tests were two-sided and we considered a significance level of 5% with no p-value adjustment for multiple comparisons.

## Results

This study included 39 children and adolescents with DTC (74.6% girls) with a mean age at diagnosis of 15 years (range 5-18). Four patients (10.3%) were under 10 years old. The demographic and clinical characteristics are shown in Table 3.

**Table 3:** Demographic and clinical characteristics RAI: radioiodine

Demographic details	n	%
Age (years)		
Median (min-max)	15 (5-18)	
Interquartile range	12-17	
Median female age (min-max)	14 (6-18)	
Median male age (min-max)	15.5 (6-18)	
Gender		
Female	29	74.6
Male	10	25.4
Histological type		
Papillary	39	100
Papillary classic variant	20	51.3
Papillary, other variants	19	48.7
Follicular variant	13	33.3
Follicular and classic variant	3	7.7
Diffuse sclerosing variant	2	5.1
Solid/trabecular variant	2	2.6
Tumor characteristics		
Solid/trabecular areas	4	10.3
Microscopic extra-thyroidal extension	22	56.4
Vascular invasion	16	43.2
Multifocal	21	53.8
Diffuse	1	2.6
Initial tumor stage		
Thyroid (N0M0)	16	41
Regional (N1a/bM0)	19	41.8
N1a	12	30.8
N1b	7	17.9
Distant (M1)	4	10.3
Metastasis at presentation		
Lung only	3	9.1
Lung, bone, and liver	1	2.6
Metastasis during follow-up		
Lung	1	2.6
Initial Surgery		
Lobectomy	4	10.3
Total thyroidectomy	11	28.2
Total thyroidectomy + lymphadenectomy	22	56.4
Completion thyroidectomy + lymphadenectomy	1	2.6
Removal of thyroglossal cyst	1	2.6
RAI therapy		
Yes	29	75.7
No	10	25.6
Number of RAI therapies (n=29)		
1	22	75.9
2	4	13.8
3	3	10.3
Total activity (mCi) (n=29)		
Median (min-max)	100 (30-220)	
Interquartile range	70-106.5	

Regarding five patients with distant disease, four had metastases discovered at post-therapy whole body scan (WBS) performed after RAIT. In the remaining one, metastatic disease was detected by a CT scan performed after one treatment with RAI with WBS showing only small cervical uptake (but non-suppressed Tg levels). Among the five patients with distant metastases, four were located in the lung only and one had them in the lung, liver, and bone (this patient had been exposed to large amounts of radiation during various cardiac catheterizations performed due to a congenital heart defect). Our cohort had no mortality or recurrence of disease (defined by “de novo” biochemical or structural evidence of disease) after a period with no evidence of disease.

Association between ATA risk groups and re-evaluation between 12-24 months of follow-up

As shown in Table 4, the majority (76.9%) of ATA low-risk patients had an excellent response at 12-24 months and 55.6% of the ATA high-risk patients had a structural incomplete response. Patients included in ATA intermediate-risk group (n=11) had a more heterogeneous response, with 36.4% having an excellent response. We found a statistically significant linear association between these two stratification systems (p=0.001).

**Table 4:** Association between ATA risk groups and re-evaluation between 12-24 months of follow-up ATA: American Thyroid Association Somers’ D (95%CI): 0.524 (0.278-0.770) Linear-by-linear test, p=0.001

	Excellent response (n=16)	Indeterminate response (n=7)	Biochemical incomplete response (n=3)	Structural incomplete response (n=7)
ATA Low Risk (n=13)	10 (76.9%)	3 (23.1%)	0 (0%)	0 (0%)
ATA Intermediate Risk (n=11)	4 (36.4%)	2 (18.2%)	2 (18.2%)	2 (18.2%)
ATA High Risk (n=9)	2 (22.2%)	1 (11.1%)	1 (11.1%)	5 (55.6%)

Association between ATA risk groups and state of disease at final follow-up

As shown in Table 5, the great majority of ATA low-risk patients (92.3%) had NED at the last medical visit, with only one (7.7%) having an indeterminate state. One-third of ATA high-risk patients had NED at final follow-up and more than half (55.5%) had persistent disease, either biochemical (11.1%) or structural (44.4%). Five ATA intermediate-risk patients (45.5%) had NED at the end of follow-up. There was a significant association between ATA risk groups and the status of the disease at the final follow-up (p<0.001).

**Table 5:** Association between ATA risk groups and state of disease at final follow-up ATA: American Thyroid Association, BED: biochemical evidence of disease, NED: no evidence of disease, SED: structural evidence of disease Somers’ D (95%CI): 0.524 (0.278-0.770) Linear-by-linear test, p<0.001

	NED (n=20)	Indeterminate (n=4)	BED (n=3)	SED (n=6)
ATA Low Risk (n=13)	12 (92.3%)	1 (7.7%)	0 (0%)	0 (0%)
ATA Intermediate Risk (n=11)	5 (45.5%)	2 (18.2%)	2 (18.2%)	2 (18.2%)
ATA High Risk (n=9)	3 (33.3%)	1 (11.1%)	1 (11.1%)	4 (44.4%)

Association between re-evaluation at 12-24 months and state of disease at final follow-up

All patients reclassified as having an excellent response at 12-24 months had NED at final follow-up; two-thirds of patients who had a biochemical incomplete response were BED at the end of follow-up; the great majority of patients with a structural incomplete response (85.7%) had SED at last medical visit (Table 6). There was a significant association between the re-evaluation at 12-24 months and the disease status at the final follow-up (p<0.001).

**Table 6:** Association between re-evaluation at 12-24 months and state of disease at final follow-up BED: biochemical evidence of disease, NED: no evidence of disease, SED: structural evidence of disease Somers’ D (95%CI): 0.782 (0.641-0.924) Linear-by-linear test, p<0.001

	NED (n=20)	Indeterminate (n=4)	BED (n=3)	SED (n=6)
Excellent response at 12-24 months (n=16)	16 (100%)	0 (0%)	0 (0%)	0 (0%)
Biochemical incomplete response at 12-24 months (n=3)	0 (0%)	1 (33.3%)	2 (66.7%)	0 (0%)
Structural incomplete response at 12-24 months (n=7)	0 (0%)	1 (14.3%)	0 (0%)	6 (85.7%)
Indeterminate response at 12-24 months (n=7)	4 (57.1%)	2 (28.6%)	1 (14.3%)	0 (0%)

The baseline ATA risk group was associated with disease status evaluated as per DRS and at the end of follow-up (in both cases p-value=0.001). Somers' D was equal to 0.524 and 0.471, respectively.

The DRS system improved the prediction of disease status at the end of follow-up by 78.2% (Somers' D=0.782, Table 6), which indicates that the DRS system is a better predictor of long-term disease status in pediatric patients compared to ATA risk stratification.

Additional treatments after DRS

Of the 16 patients who had an excellent response to initial treatment, 11 (68.8%), underwent a single RAI administration with no need for additional treatments. Of the three patients who had a biochemical incomplete response, one was initially classified as ATA high-risk and underwent three RAI administrations due to lung uptake in the first two. Of the seven patients with structural incomplete response, one (14.3%) had a second surgery due to the persistence of nodal disease in a post-surgical neck ultrasound. Notably, five of these patients (71.4%) had more than one RAI administration (three patients received two RAI administrations, and two patients received three). Of the seven patients with indeterminate responses, one (14.3%) underwent a second surgery due to persistent nodal disease, and six patients (85.7%) received RAI.

Factors associated with persistent disease at 27 months (minimum follow-up for all patients)

At 27 months of follow-up, patients were divided into two groups: a "disease-free" group which included patients with NED (n=20), and a "persistent disease" group with patients with BED and SED (n=11). Only two patients had a different disease status at 27 months compared to the final follow-up. Both had BED at 27 months which turned into an indeterminate state at the time of the last medical visit. In Table 7, we show by univariate analysis, that factors significantly associated with the persistence of disease at the end of follow-up were male sex (OR=8.75, p=0.009), the presence of lymph node (OR=27.84, p=0.001), distant metastases (OR=24.60, p=0.006) at diagnosis, extra-thyroidal extension (OR=7.00, p=0.024) and higher values of the sTg (with a 1% increase in the odds of persistent disease per unit increase in sTg, p=0.049).

**Table 7:** Factors associated with persistence of disease at 27 months BED: biochemical evidence of disease, NED: no evidence of disease, SED: structural evidence of disease, Tg: thyroglobulin *Two patients with missing information concerning vascular invasion at diagnosis **Interfering antithyroglobulin antibodies (TgAB) for three patients with persistent disease and three disease-free patients; five patients in NED did not receive radioiodine therapy (RAIT)

Variables	Disease free (NED, n=20) n (%)	Persistent disease (BED+SED, n=11) n (%)	Odds Ratio (95%CI)	p-value
Sex				0.009
Female	18 (90%)	5 (45%)	1	
Male	2 (10%)	6 (55%)	8.75 (1.68-60.29)	
Age, years old				0.302
Median (P25-P75)	13.5 (12.8-16)	14 (10.5-16.5)	0.89 (0.71-1.11)	
Tumor size, cm				0.492
Median (P25-P75)	1.95 (1.45-3.13)	2.90 (2.50-3.75)	1.16 (0.74-1.90)	
N status				0.001
N0	11 (55%)	0	1	
N+	9 (45%)	11 (100%)	27.84 (2.92-3748.54)	
N1 subclassification				1.000
N1a	3 (33%)	3 (33%)	1	
N1b	6 (67%)	6 (67%)	1.00 (0,15-6.55)	
M status				0.006
M0	20 (100%)	7 (64%)	1	
M1	0	4 (36%)	24.60 (2.22-3412.56)	
Extra-thyroidal extension				0.024
No	10 (50%)	1 (9%)	1	
Yes	10 (50%)	10 (91%)	7.00 (1.27-73.48)	
Vascular invasion*				0.210
No	11 (61%)	4 (36%)	1	
Yes	7 (39%)	7 (64%)	2.56 (0.59-12.11)	
Multicentric tumor				0.513
No	8 (40%)	3 (27%)	1	
Yes	12 (60%)	8 (73%)	1.65 (0.37-8.35)	
Stimulated Tg, ng/ml**				0.049
Median (P25-P75)	0.95 (0.38-5.43)	63.9 (27.0-224.8)	1.01 (1.00-1.05)	

Of the 20 patients in whom was possible to evaluate sTg, one had indeterminate disease status, eight had persistence of disease, and 12 were disease-free with medium values being 63.9 ng/ml and 0.95 ng/ml, respectively (Table 6). Although the sample size is not robust, we conducted an exploratory ROC analysis to determine the cut-off value of sTg that could better differentiate patients with persistent disease from those without disease or indeterminate status. The area under the curve (AUC) was 0.901 (95% CI 0.767-1.000) and the optimal threshold to discriminate patients with persistent disease was 11.5 ng/mL, with a sensitivity of 87.5% and a specificity of 83.3%. If we exclude from the analysis the two patients that had BED at 27 months but were indeterminate at the last follow-up, that cut-off value changes to 17.85 ng/mL with both sensibility and specificity of 83.3% (AUC: 0.896 (95% CI 0.750-1.000)).

## Discussion

In our series of 39 DTC pediatric patients, there is a female preponderance concordant with the literature [2,9]. The proportion of disease confined to the thyroid (41%), regional disease (48%), and distant metastases at diagnosis (10.3%) meet the findings of a multicentric study performed by Hogan et al [9]. Around 60% of our patients with pulmonary metastases had regionally extensive disease, a finding supported by others [3], emphasizing the potential role of lymph node status at diagnosis for the prognosis. The DRS system was evaluated in pediatric patients by others [4-7,10]: Sung et al. [7] found that both ATA risk stratification and DRS systems were associated with the clinical outcomes (p<0.001 for both); Zanella et al [4] showed that DRS was an excellent tool for predicting clinical outcomes in their cohort of sixty-six patients, in which the majority remained in the same category they had in DRS; Kim et al. [5] (128 patients) compared ATA risk stratification and DRS system and demonstrated the superiority of the latter in predicting disease outcomes, similarly to Adjari et al. [10] and Lazar et al [6].

In our study, there was also a significant association between ATA group risks, DRS, and the state of disease at the final follow-up. However, the association of DRS to the status of the disease at final follow-up is stronger than the association between ATA group risks and the final clinical outcome. Indeed, the assessment of the response to treatment at one to two years can dramatically change the prognosis in an ATA high-risk patient that exhibits an excellent response to treatment and of a patient initially classified as ATA low-risk that has a biochemical or structural incomplete response to treatment. In our cohort, all ATA high-risk patients who had an excellent response to treatment had NED at the final follow-up. The DRS had notable importance also in the ATA intermediate-risk patients, in whom there was a similar percentage of patients with NED (n=5; 45.5%) and with persistent disease (n= 4; 36%). Reclassification of these patients after treatment was highly concordant with final outcomes: 100% of those with an excellent response to treatment had NED and 75% of patients who had biochemical or structural incomplete responses to treatment had persistence of disease. These findings emphasize the importance of adding variables that take into account the response to treatment rather than using a static stratification based only on histopathological data in order to plan management in these patients better. This is even more relevant in the pediatric population, who often have stable disease despite initial signs of aggressiveness, and in whom there is a particular concern in reducing potential complications from the iodine therapy, like the apparent increased risk of second malignancies [3].

Interestingly, in the seven patients with an indeterminate response to therapy, most (n=4; 57.1%) had NED at the end of follow-up, two (28.6%) were still indeterminate and one (14.3%) was reclassified as having BED, with none of the patients having SED. This data reinforces the indolent nature of DTC in the pediatric population. Factors significantly associated with persistent disease at 27 months were: male sex (OR=8.75, p=0.009), the presence of lymph node (OR=27.84, p=0.001), distant metastases (OR=24.60, p=0.006) at diagnosis, extra-thyroidal extension (OR=7.00, p=0.024) and higher values of the sTg. In our series, this male preponderance may be explained by the higher frequency of metastases at diagnosis in males (p=0.036). The presence of nodal disease has been associated with persistent disease in other studies [5,7], and Pires et al. [10] also found that female gender and distant metastasis were significantly associated with disease-free status. Of note, ATA risk group stratification and DRS were two other factors significantly associated with persistent disease, confirming the findings of others [4]. Regarding sTg, our work showed an optimal threshold of 11.5 ng/mL to discriminate patients with persistent disease from patients in remission at the end of follow-up. The usefulness of sTg has been addressed by others [4,11]. Liu et al. [11] found a cut-off value of 17.8 ng/mL and Zanella et al. [4] determined a cut-off point of 37.8 ng/mL to predict persistent disease. However, when we exclude the two patients that were BED in 27 months but had an indeterminate status, the cut-off value changes to 17.85 mg, that, in accordance with the findings of others, is higher than the value calculated for the adult population in a meta-analysis [12] and these findings may reflect the higher aggressiveness of DTC in the pediatric population [11]. sTg might have a role in the prognostication of these patients, but more studies are needed to determine specific cut-offs in these patients.

Strengths and limitations

Our study has some notable limitations. First, the follow-up time was relatively short, which may account for the large group of patients with an indeterminate state at the last medical visit, the absence of cases of recurrent disease, the strong association between revaluation between 12-24 months, and the state of disease at final follow-up. Second, because some of these patients went to surgery in other institutions, we might have missed information about disease presentation and the initial therapy. However, this limitation was attenuated by the fact that all histological reports were reviewed, and all the patients had a post-operative cervical ultrasound performed in our institution. The follow-up was uniformly pursued after referral by a multidisciplinary team of experts in thyroid oncology. Third, a potential confounding factor for the effectiveness of initial therapy is the fact that some patients repeated surgery and/or RAITs before the revaluation time at 12-24 months, while some repeated RAI administrations after this time period.

## Conclusions

To our knowledge, the present study is the first to evaluate the DRS system in a pediatric population with DTC in Europe and to emphasize that, although both ATA risk stratification and DRS are suitable for the evaluation of the risk of recurrent /persistent disease, the latter stratification system can refine the initial predictions of the ATA risk groups. Therefore, we think that DRS should be incorporated as a tool in the management of DTC in children, as it has been for the adult population.
